# Biomechanics of extra-alveolar mini-implants

**DOI:** 10.1590/2177-6709.24.4.093-109.sar

**Published:** 2019

**Authors:** Marcio R. Almeida

**Affiliations:** 1 Universidade Norte do Paraná, Curso de Mestrado Acadêmico em Ortodontia e Doutorado em Dentística (Londrina/PR, Brazil).

**Keywords:** Orthodontic miniscrews, Mini-implants, Class I malocclusion, Class II malocclusion, Class III malocclusion

## Abstract

It is undeniable that extra-alveolar mini-implants anchorage has revolutionized Orthodontics. Correspondingly, the proper understanding of mini-implants biomechanics allowed to broaden the range of dental movements as never seen before in clinical practice. However, in order to produce better treatments, especially regarding the effects in occlusal plane, it is important to be aware of the numerous possibilities of applying force systems based on skeletal anchorage. Thus, this paper aims to address, by means of clinical cases, the application of biomechanics concepts that are extremely relevant to the proper employment of extra-alveolar mini-implants.

## RESUMO

É inegável que a ancoragem com mini-implantes extra-alveolares revolucionou a nossa especialidade. No mesmo sentido, o correto entendimento da biomecânica com mini-implantes permitiu ampliar a gama de movimentos dentários como nunca visto na prática clínica. Deve-se, porém, atentar às diversas possibilidades dos sistemas de aplicação das forças decorrentes do uso da ancoragem esquelética, com vistas a produzir tratamentos cada vez mais eficientes, principalmente em relação aos efeitos no plano oclusal. Assim, o presente artigo versa, por meio de casos clínicos, sobre a aplicação de conceitos da biomecânica que são extremamente relevantes para o correto emprego dos mini-implantes extra-alveolares.

## INTRODUCTION

Ever since skeletal anchorage became part of orthodontists' treatment plan, more specifically orthodontic mini-implants, encouraging results are being achieved regarding anchorage. Mini-implants, or miniscrews, figure as an absolute anchorage system of great utility in orthodontic practice. Although it is common to install them in areas of the alveolar process located between the roots of contiguous teeth, new sites, referred to as extra-alveolars, have been suggested.^1^ ^-^ ^6^ Numerous authors^7^ ^-^ ^15^ recommend the infrazygomatic crest and the mandibular buccal shelf area as suitable places for a great number of orthodontic therapies that require an efficient and safe anchorage system, broadening treatment limits, due to the following benefits:

1) Less risk of damaging roots.
2) Larger quantities of cortical bone in insertion points, which allows the use of mini-implants with larger diameter (2 mm) and greater length (12/14 mm).
3) They do not interfere with mesiodistal movement of teeth or groups of teeth.
4) The achieved anchorage is appropriate for the retraction or mesial movement of the entire dental arch, allowing simultaneous movement of the entire dentition.
5) Low failure percentage, if compared to the conventional mini-implants.
6) Smaller number of mini-implants used to solve complex cases.

However, it is important to reflect on a pivotal question regarding extra-alveolar mini-implants: Why is this mechanics revolutionizing Orthodontics? The direct answer is detailed below:

1) Low-cost and simple installation.
2) Allows to treat complex issues, which were restricted to miniplates treatment.
3) Allows to apply multi-vector forces, enabling treatment for a variety of problems.
4) Allows to modify the incisal/occlusal plane.
5) Allows to treat related issues.

Consequently, the aim of this article is to depict the importance of a detailed analysis of the biomechanics system force generated by the application of extra-alveolar mini-implants in the infrazygomatic crest or buccal shelf areas, especially in regards to the changes and control of occlusal planes.

## INDICATIONS 

Despite many indications, in comparison to intra-alveolar mini-implants, the ones installed in the infrazygomatic crest (IZC) or buccal shelf (BS) areas, referred to as extra-alveolars, are most commonly used for distalizing the whole maxillary and mandibular dentition - because they allow better anchorage immediately after insertion (primary stability) into these reinforced maxillary and mandibular bone areas. 

**Figure 1:**
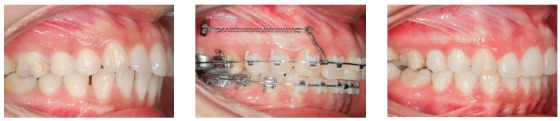
Clinical case with indication of mini-implants in the IZC for dentoalveolar *en masse* retraction of the entire maxillary arch.

**Figure 2:**
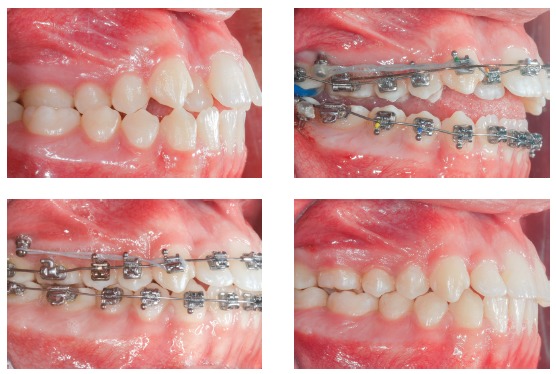
Distalization made through canine and premolar sliding, in order to obtain anterior space, in a patient with severe maxillary crowding.

**Figure 3:**
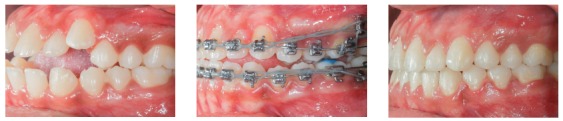
Posterior teeth intrusion, associated to retraction of the entire maxillary arch.

**Figure 4:**
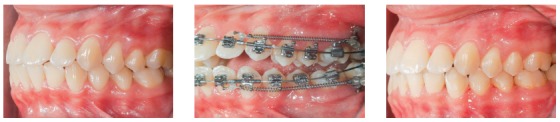
*En masse* retraction with mini-implants in the IZC and BS to reduce Class I biprotrusion.

**Figure 5:**
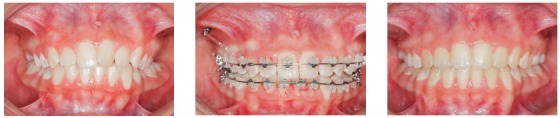
Asymmetrical clinical case (roll, transverse plane rotational axis): oblique line of force action, from the mini-implant in the right IZC to the arch, allows roll axis asymmetry correction and simultaneous correction of the midline.

**Figure 6:**
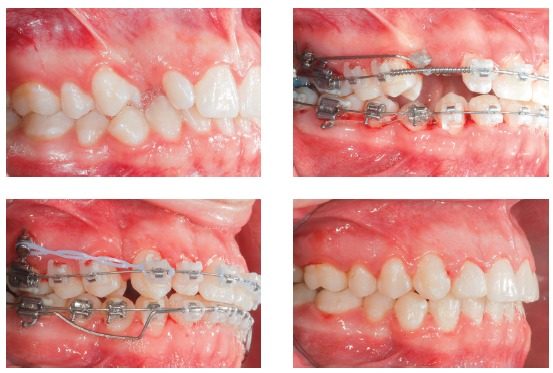
Clinical case of impacted maxillary right canine, treated with extra-alveolar anchorage supporting a cantilever for canine traction.

**Figure 7:**
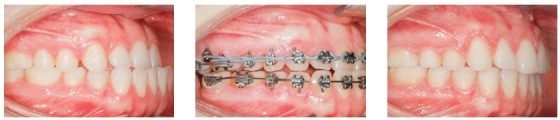
Orthodontic-surgical case preparation by means of mini-implant, in order to accelerate Class III decompensation and maxillary teeth retraction.

Mini-implants in the IZC are recommended for the following cases: maxillary anterior teeth retraction, performed by segments or dentoalveolar *en masse* retraction of maxillary arch (Fig 1); canines and premolars distalization with sliding mechanics, in order to obtain anterior space (Fig 2); posterior teeth intrusion associated to retraction of the entire dental arch (Fig 3); patients requiring retraction of segments of teeth, in order to correct dental protrusion (Fig 4). Other indications for mini-implants usage in the IZC are: asymmetry correction of the occlusal plane and midline deviation (Fig 5); anchorage for cantilever use in impacted canine traction (Fig 6); orthognathic surgery preparation in Class III cases (Fig 7).

**Figure 8:**
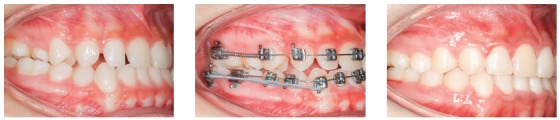
Class III compensatory treatment (camouflage) with mini-implants in BS and entire mandibular arch retraction.

**Figure 9:**
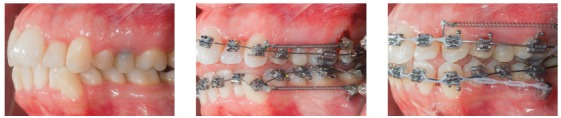
Excessive anterior mandibular crowding case, treated with canine distalization by means of sliding mechanics, using mini-implants in the BS.

**Figure 10:**
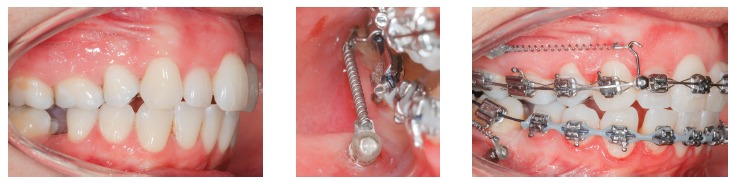
Mandibular teeth mesialization mechanics, using mesioangulated mini-implant in the BS, associated to entire maxillary arch retraction with mini-implants in the IZC.

**Figure 11:**
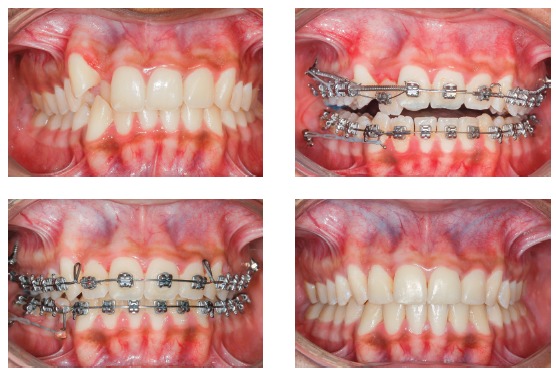
Mandibular midline asymmetry correction, with the aid of one mini-implant in the right BS.

**Figure 12:**
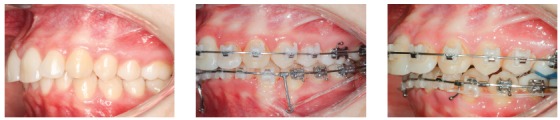
Class II orthodontic-surgical case, in which retraction mechanics of the entire mandibular arch was used, in order to create overjet for future mandibular advancement.

Indications for mini-implants placed in the mandibular BS area are quite similar to mini-implants in the IZC, in other words, they can be used in case of: Class III compensatory treatment (Fig 8); retraction and/or canine distalization in cases of excessive mandibular crowding (Fig 9); mesial movement of molar (Fig 10); intrusion of posterior teeth; correction of occlusal plane asymmetries and midline deviation (Fig 11); anchorage for cantilever use in mandibular impacted canine traction; orthognathic surgery preparation in Class II cases (Fig 12).

## EMPLOYED FORCE MAGNITUDE

The force magnitude used in extra-alveolar mini-implants mechanics is an important factor for the therapy's success, due to its influence on anchorage stability^2^ ^-^ ^9^. The recommended magnitude varies from 220 to 340g (8 to 12 oz) for mechanics with mini-implants in the IZC area, and from 340 to 450 g on the ones with mini-implants in the BS area. It is worth mentioning that such force magnitude enables distalization of the entire arch, in other words, *en masse* retraction. For cases in which a partial retraction is needed - for example, to retract canines and premolars -, force must be adjusted between 150 and 200 g.

## BIOMECHANICS IN THE USAGE OF EXTRA-ALVEOLARS MINI-IMPLANTS

**Figure 13:**
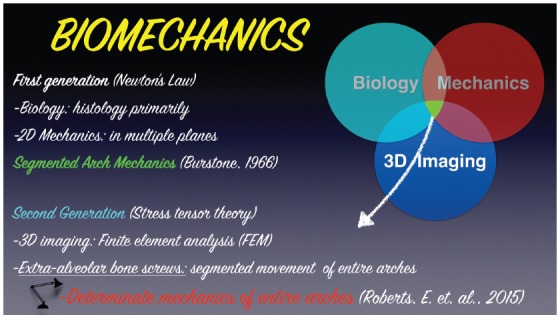
Scheme illustrating the transition from the first generation (2D) of classic Biomechanics to the second generation (3D), with finite elements, and an analysis of stress levels as well as periodontal ligament tension in determinate mechanics, using extra-alveolar mini-implants.

One of the most important aspects when using extra-alveolar mini-implants is applying the best force system. Nanda,^16^ in 2010, developed the term “family of biomechanics", which, by definition, are the biomechanical concepts critical for understanding treatment of numerous issues, regarding force systems and the resultant force for a required motion. This author declared the importance of understanding the force-moment, its magnitude, direction and duration, point of application and prediction of center of resistance, center of rotation, differential moment, etc. The requirements for a clinical application consistent with Biomechanics include basic information that are necessary for understanding the physics principles that are common to every orthodontic equipment. Thus, the first generation of Biomechanics (Fig 13), based on the understanding of bidimensional systems (2D) in accordance to Newton's laws, correspond to the classical segmented arch mechanics.^17^ However, presently, we now proceed to the second generation of Biomechanics,^18^ with finite elements (3D) study, in order to determine the correct stress in the periodontal ligament (determinate mechanics) (Fig 13). In this manner, with the intention of simplifying the application of Biomechanics concepts for treating complex malocclusions, instead of segmented mechanics and utilizing the numerous accessory devices from the segmented arch's classical technique, it is possible to apply multivector mechanics provided by a sophisticated force system using extra-alveolar mini-implants. Thus, the biomechanics principles (force systems) applied for the use of extra-alveolar mini-implants will be detailed further ahead.

### Biomechanics of mini-implants in the BS

**Figure 14:**
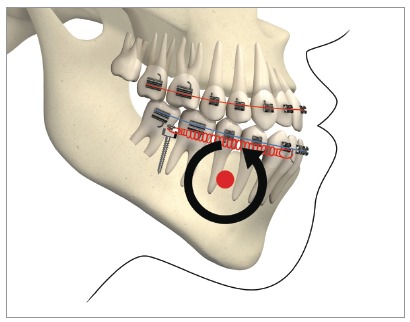
Determinate mechanics, according to Roberts et al.^18^, stems from retraction mechanics of the mandibular dentition, produced by two mini-implants in the BS and full-size rectangular arch with NiTi springs, applying 200g of constant force, in Class III patients. Source: Almeida et al.^3^

With the exception of the need for third molar extraction, this mechanics is considered non-extraction and non-surgical, and allows the entire mandibular dentition to be retracted in one block, since the mini-implants are located outside the roots' line of action and, therefore, do not interfere with the movement of the entire arch. Roberts et al^18^ demonstrated, through finite elements (3D) and CBCT analysis, a robust system, considered “statically determinate”, which stems from the mechanics for retraction of the entire mandibular dentition, produced by the use of two mini-implants in the BS and a full-size rectangular archwire, with NiTi springs applying constant force of 200 g (Fig 14). The authors also alluded to the existence of three critical factors for the mechanics to be considered statically determinate and able to be studied by means of finite elements: 1) use of rectangular arch (full-size) with torque control during retraction; 2) relative constant force stemming from superelastic NiTi springs; and 3) force applied directly to the arch. Thus, the concept of “segment” arises, which is defined in the sagittal plane as a block of teeth, with a rectangular archwire inserted in all anterior teeth, for torque control, which allows the entire arch to be retracted *en masse*, with no significant inclination.^18^

**Figure 15:**
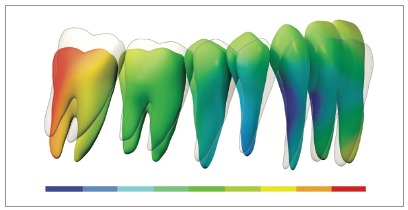
Scheme showing, by means of finite elements, mandibular posterior rotation over a rotational axis in the canine area. Source: Roberts et al.^18^

Roberts et al^18^ consider this system for mandibular teeth retraction anchored on two mini-implants in the BS to be an excellent resource for a conservative and non-extraction treatment of Class III malocclusion with anterior open bite, since the retraction force in the entire arch generates an intrusive force in the molars and an extrusive force in the incisors, caused by the rotation of the mandibular arch. This counterclockwise rotation of the mandibular plane (Fig 14), observed through finite elements analysis, resulted in a 3-mm molar intrusion and 2-mm incisor extrusion, favoring open bite closure and simultaneous Class III correction.^18^ In the superimposition of mean effects, during finite elements analysis, an axis of rotation of the entire arch was observed close to the mandibular canine area (Fig 15).^18^

**Figure 16:**
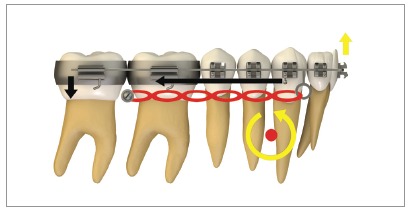
Scheme illustrating the application of mini-implants mechanics in the BS, in order to retract the entire mandibular dentition in a single block. Counterclockwise rotation of the mandibular occlusal plane can be seen, due to the force action line being placed occlusal to the mandibular center of resistance. Thus, the generated moment causes incisor extrusion and molar intrusion.

Figure 16 illustrates the mechanics with mini-implants in the BS used for single-block retraction of the entire mandibular dentition. Shih et al^19^ demonstrated mandibular occlusal plane rotation during mandibular teeth retraction with BS mini-implants and traction force from the archwire to the mini-implant. This counterclockwise rotation occurs because the force action line is located occlusal to the center of resistance of the arch; consequently, it generates a moment that causes incisor extrusion and molar intrusion. Arch rotation associated with retraction manifests with clear distal inclination of the molars. A decrease in mandibular plane angle can also be noted.^19^

**Figure 17:**
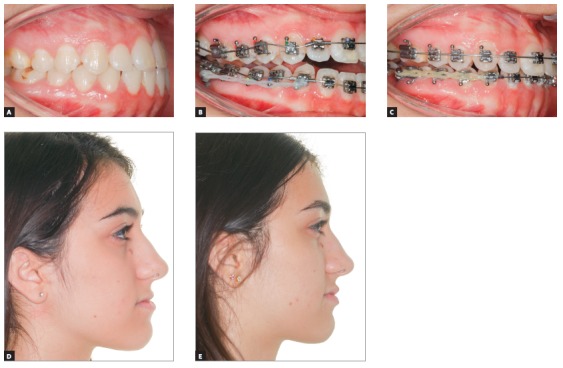
Initial photographs: A) Without brackets, and B) with brackets from second molar to second molar, occlusal build-up and retraction biomechanics of mandibular teeth. Mechanics with mini-implants in the BS for *en masse* retraction of mandibular dentition, in patient with Class III malocclusion and retreatment. A mandibular CuNiTi 0,014 x 0,025-in archwire was used. This mechanics lasted four months. In C, the sagittal relationship correction and improvement in the vertical direction can be observed, caused by the rotation of the occlusal plane, due to the force action line being occlusal to the center of resistance of the arch. Thus, a moment is created, causing incisor extrusion and molar intrusion. In D and E, the comparison between the initial profile and after four months of treatment: there is a discernible improvement in the facial profile, especially in the lower lip, which was retracted after mandibular incisor lingual movement.

Figure 17 shows the mechanics for mandibular dentition *en masse* retraction during retreatment of a patient with Class III malocclusion. Mandibular teeth retraction phase was simultaneously started along aligning and leveling, anchored on a mini-implant adapted to the BS and chain elastics connecting the mini-implant to the mandibular canine, locked by two stops. A 0.014 x 0,025-in CuNiTi mandibular archwire was used. Other details of this mechanics, which lasted for approximately four months, will be given further in the paper. In Figure 17C, correction of the sagittal relation and improvement of the vertical dimension can be observed as a result of the occlusal plane rotation, which happens because the force line of action is occlusal to the center of resistance of this arch; thus, generating a moment, and causing incisor extrusion and molar intrusion. Figures 17D and 17E show a clear improvement in facial profile after four months of treatment. There is an evident change, especially in the lower lip, which was retracted as a consequence of lingual inclination of the mandibular incisors.

### Biomechanics of mini-implants in the IZC

**Figure 18:**
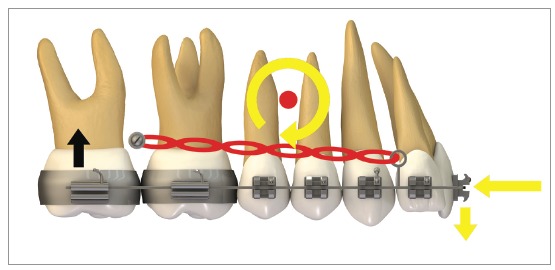
Scheme illustrating the application of mini-implants mechanics in the IZC to retract the entire maxillary dentition in a single block: a clockwise rotation in the occlusal plane can be observed, due to the force action line being occlusal to the center of resistance of the arch. Thus, a moment is generated, which leads to incisor extrusion and molar intrusion.

**Figure 19:**
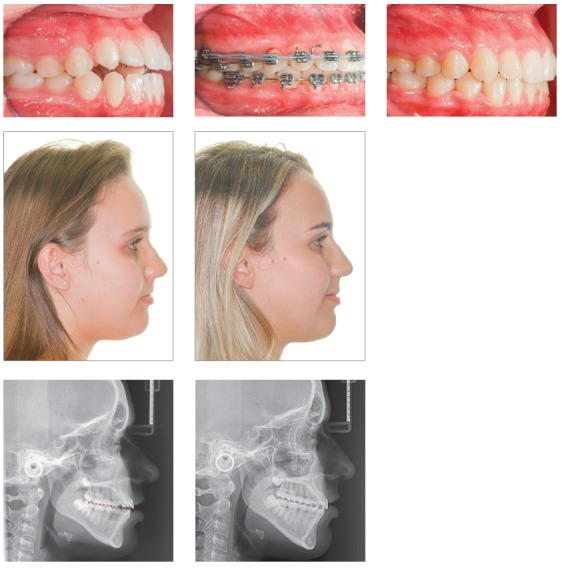
Clinical case of patient with Class II malocclusion and anterior open bite, treated with two mini-implants in the IZC. Clockwise rotation of the occlusal plane around the Cr favored open bite closure, as well as simultaneous Class II correction.

Similar to the biomechanics of mini-implants in the BS, mini-implants in the IZC generate a retraction force system during distalization of the entire maxillary arch. The retraction force in the entire maxillary arch generates intrusive force in the molars and extrusive force in the incisors, caused by clockwise rotation around the center of resistance (Cr) of the entire maxilla, which is located between the premolars (Fig 18). The force line of action passes bellow (occlusal) the maxillary Cr and, consequently, causes this rotation. For that reason, incisor extrusion must be expected, which may be unfavorable for patients with deep bite. On the other hand, this occlusal plane clockwise rotation favors simultaneous open bite closure and Class II correction (Fig 19). Retraction biomechanics can be modified through changes in the height of hooks in the anterior area and in the force line of action. More details about this mechanics will be given further ahead.

## GENERALITIES OF THE EXTRA-ALVEOLAR MINI-IMPLANTS' FORCE SYSTEMS

The generalities of the force systems stemming from usage of mechanics with extra-alveolar mini-implants are highlighted below.

**Figure 20:**
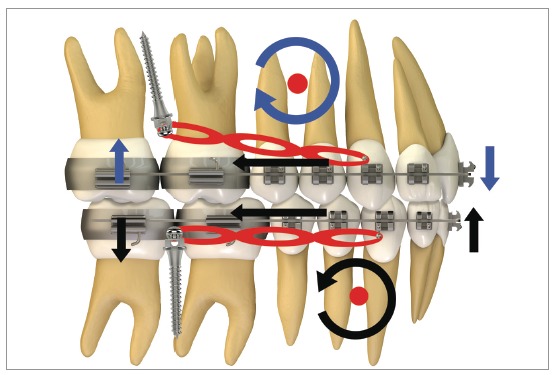
Scheme illustrating the application of a mechanics with mini-implants in the IZC and BS for retraction of the entire dentition, maxillary and mandibular, in a single step. It can be observed that counterclockwise rotation in the mandibular occlusal plane happens because the force action line is occlusal to the arch's center of resistance; generating a moment, which leads to incisor extrusion and molar intrusion. In a similar manner, a clockwise rotation occurs in the maxilla due to the force action line being occlusal to the arch's center of resistance, thus, a moment is generated, causing incisor extrusion and molar intrusion.

Figure 20 illustrates, in a general way, the biomechanics for distalization of the whole dentition, maxillary and mandibular, in a single step. It can be observed that, during *en masse* teeth retraction, a clockwise moment is created in the maxilla and a counterclockwise one, in the mandible. These moments of resultant force promote uncontrolled inclination movement in the posterior teeth since the direction of force passes away from the center of resistance (Cr) of the maxilla and mandible. Furthermore, vertical forces are generated over incisors and molars. With this mechanics, incisors present extrusion, increasing overbite, while molars respond with an intrusive force that tends to open the bite in posterior area. 

**Figure 21:**
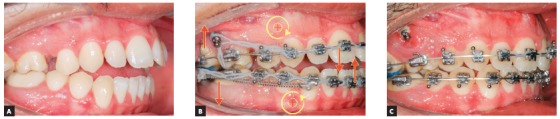
A, B) Patient with biprotrusion and anterior open bite, treated with mini-implants in the IZC and BS for distalizing mechanics. B) Curved yellow arrows represent moments generated around the maxilla and mandible's center of resistance, causing occlusal plane rotation (clockwise for the maxilla and counterclockwise for the mandible). Vertical red arrows demonstrate extrusion vectors over the incisors and intrusion vectors over the molars. In C, after four months of treatment, an improvement can be seen in the anterior vertical relationship. A clear uprighting of the incisors can be expected.

This sophisticated force system ends up modifying the maxillary and mandibular occlusal planes. Figure 21 shows the force system for maxillary and mandibular dentoalveolar distalization supported by mini-implants located in the IZC and BS areas. It can be noted there is occlusal plane rotation, which assists in treating anterior open bite as well as dentoalveolar protrusion.

### Force systems variations

**Figure 22:**
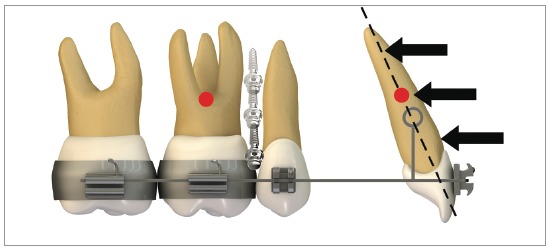
Scheme exemplifying how to modify the force action line when using extra-alveolar mini-implants: the mini-implants' installation height can be changed or the height of hooks in the anterior area can be altered.

Thus, it can be observed that understanding concepts related to Biomechanics is crucial for treatments that utilize extra-alveolar mini-implants. As mentioned before, each particular case requires correct force application (direction and anchoring point). For that reason, there are two important factors to be taken into account when studying correct force design, in which different types of dental movements can be obtained^20^ (Fig 22): 1) height of hooks in the anterior area; 2) height modification in extra-alveolar mini-implants insertion.

**Figure 23:**
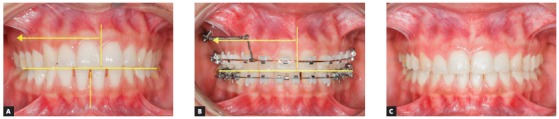
A) Clinical case demonstrating a sagittal occlusal relationship between the arches' in unilateral Class II molars, in which there were a complete Class II on the right side and a Class I relationship on the left side. B) The maxillary dental midline was deviated 3mm to the left in relation to the middle sagittal plane, while the mandibular dental midline was centralized. The chosen treatment was by the use of mini-implant in the IZC and asymmetric mechanics for unilateral distalization. The system was composed of rectangular 0,017 x 0,025-in steel archwire, used to adapt a modified hook with greater cervical extension (15 mm) in order to have the force action line as close as possible to the center of resistance (Cr) of all teeth and a translation movement of the midline could be obtained. The mini-implant is located in the same line as the modified hook, mesial to the canine. Because the patient presented a symmetrical mandibular occlusal plane (red dotted line) in relation to the frontal occlusal plane, thus, in order to prevent any inclination, a force parallel to the occlusal plane was required. C) Case completion.

It is not always possible to change mini-implants installation height because there are numerous factors that influence the choice of the ideal place. However, given the force direction that is required for each case, it is known that the type of anterior hook/power arm, regarding its height and location, will be a decisive factor for the expected type of movement. Changes in force geometry through different hooks or power arm in the anterior area of the arch can influence incisor torque control, as well as vertical changes occurring in the area (open bite or deep bite)^20^. Furthermore, the use of asymmetrical forces to correct Class II subdivision, by means of mini-implants in the IZC, must take into consideration the possible occlusal plane inclination. In a similar manner, asymmetrical treatments of midline deviations must be extremely well planed, regarding the correct application of the forces' line of action (Fig 23). 

Clinically, when utilizing mechanics with extra-alveolar mini-implants, such differential movements can be reproduced on the anterior teeth by modifying the force line of action, through changes in hooks/power arms length.

#### Use of short hook

**Figure 24:**
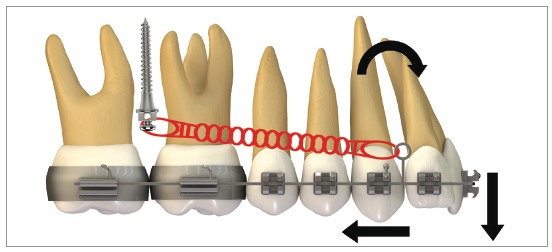
Use of short hook during retraction of the entire arch with 350g/side force stemming from a NiTi spring connected from the mini-implant to the short hook attached to the archwire. The force passes under the Cr; which means the anterior teeth are likely to rotate clockwise (curved arrow), losing torque and generating a vertical extrusion force upon the incisors.

**Figure 25:**
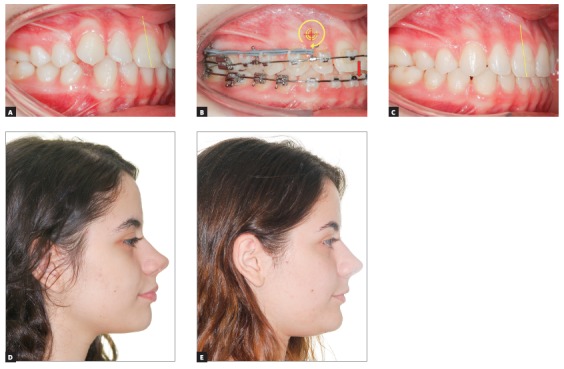
A) Patient with Class II malocclusion treated with mechanics for distalizing the entire maxillary arch, using mini-implant in the IZC. B) Force action line passes bellow the anterior teeth's Cr. When applying distalizing force in the entire maxilla, due to the oblique force (bellow the Cr), the anterior teeth tend to rotate clockwise (curved yellow arrow), losing torque, while a vertical extrusion force occurs upon the incisors (red arrow). C-E) A good maxillomandibular relationship was achieved, as well as in the vertical direction. Complete treatment lasted 24 months.

Figure 24 shows a force system in which an anterior retraction is promoted by the force generated by a NiTi spring or chain elastics, connecting the mini-implant to a short hook attached to the archwire. Anterior teeth have a tendency to rotate clockwise when retraction force is applied by means of a force that passes below the Cr (Fig 24, curved arrow), which leads to torque loss and a vertical extrusion force on the incisors. Clinical case in Figure 25 illustrates the application of a distalizing mechanics in the entire maxillary arch, by means of mini-implants in the IZC, in which force action line passes below the anterior teeth's Cr. Anterior teeth are likely to rotate clockwise when distalization force is applied to the entire maxillary dentition (Fig 25, yellow curved arrow), which causes torque loss, and creates a vertical extrusion force upon the incisors (Fig 25, red straight arrow). After appliance removal, a clear incisor inclination to lingual can be seen.

#### Use of middle length hook

**Figure 26:**
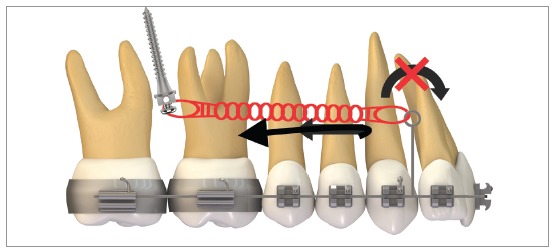
Scheme illustrating a biomechanics for retraction of the entire maxillary arch with a middle length hook: height of the hook positioned mesial to the canine allows the force action line to pass at the height of incisors' center of resistance.

When retracting the entire dental arch, if there is intention of preserving the anterior torque, a change in the geometry of force direction must be made. The example on Figure 26 shows the exact solution on how to keep torque during distalization of the entire arch: height of the hook mesial to the canine was increased, allowing the force action line to pass close to the incisor's center of resistance. Anterior moment is likely to be canceled out because of this procedure - as can be seen in Figure 26- and, during retraction, incisor torque can be maintained, with less change in the occlusal plane.

**Figure 27:**
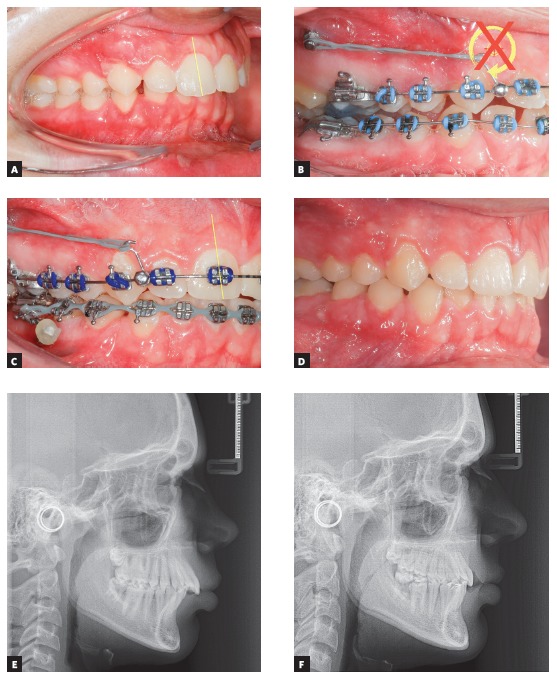
A) Patient with Class II malocclusion and overbite, treated with mechanics for distalizing the entire maxillary arch, using mini-implants in the IZC. B) Force action line passes over the anterior teeth's Cr. By applying distalization force in the entire maxilla, using force parallel to occlusal plane (Gurin on the same level as the mini-implant), the anterior teeth usually keep their initial inclination (C), occurring no vertical force. D) Image illustrating case conclusion. E, F) Initial and final lateral teleradiographies.

The clinical case in Figure 27 illustrates the application of a mechanics to distalize the entire maxillary arch, for Class II malocclusion treatment using mini-implant in the IZC and chain elastics. The force action line is passing over the anterior teeth's Cr, due to the middle positioning of the Gurin in relation to the mini-implant. When distalization force is applied to the entire maxilla, with force parallel to the occlusal plane, anterior teeth are likely to keep their initial inclination, minimizing vertical forces. 

#### Use of long hook

**Figure 28:**
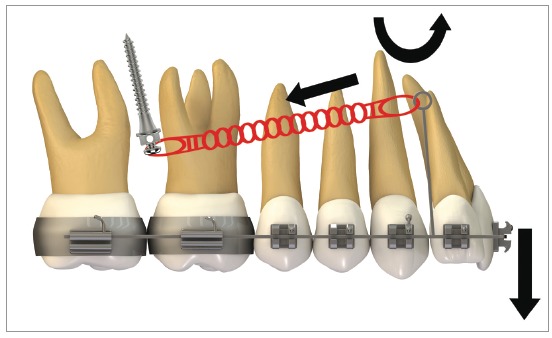
Scheme illustrating a case with maxillary premolar extraction and the biomechanics for anterior retraction: height of the hook mesial to the canine allows the force action line to pass above the incisors' center of resistance. This procedure generates a anterior counterclockwise moment during simultaneous incisor retraction and extrusion. However, it is important to point out that this procedure may be more difficult to be done at the clinic, due to the possibility of injuring patient's oral mucosa.

In order to provide proper lingual root torque to the incisors, during distalization of the entire arch, hook/power arm length must be extended, in order to make the force pass above the center of resistance, generating a counterclockwise moment on these teeth. Park et al^21^ alluded to the fact that, during retraction of the entire maxillary dentition, archwires with long hooks must be used, to create lingual root torque on the incisors. Figure 28 shows retraction mechanics with counterclockwise moment (lingual root torque) and extrusion effect on incisors.

**Figure 29:**
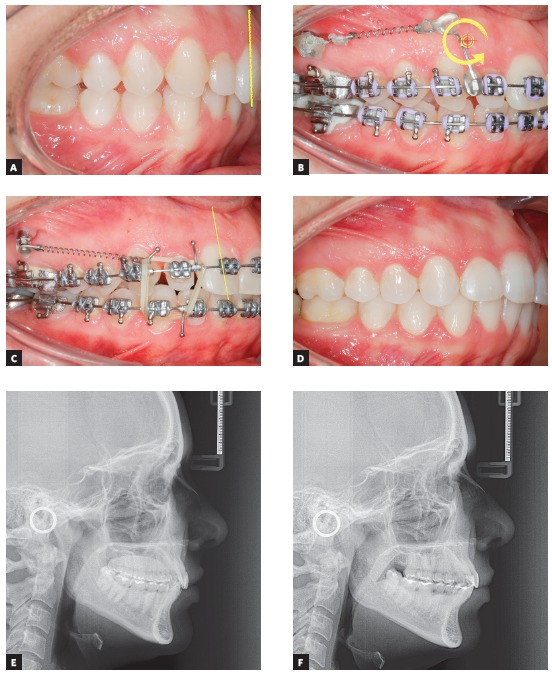
A) Patient with Class II malocclusion, division 2, and overbite, treated with mechanics to distalize the entire maxillary dentition, using mini-implant in the IZC. B) A power arm made of TMA 0,017 x 0,025-in wire, supported by a a longer criss-cross tube, was used to elevate the force above the incisors' Cr. A counterclockwise moment can be observed, which will affect the incisors' torque. In C, a great torque control of the anterior teeth can be seen. D) Resin build-up was performed in the lateral incisor, due to a Bolton discrepancy. E, F) Initial and final lateral teleradiographies.

The clinical case on Figure 29 shows a Class II division 2 malocclusion, in which distalizing mechanics was applied, giving priority to anterior torque: it can be noted that height of the hook positioned mesial to the canine allows force action line to pass above the incisors' center of resistance. A power arm made with 0,017 x 0,025-in TMA wire, supported by a a long criss-cross tube, was used to raise force above the Cr. This procedure generates a counterclockwise anterior moment during retraction and a simultaneous extrusion of the incisors. It is important to point out that, during clinical practice, such situation may be more complicated due to the possibility of injuring the patient's oral mucosa.

When using extra-alveolar mini-implants, understanding Biomechanics is a crucial step for a good treatment and successful outcome of clinical cases. A recently published article on extra-alveolar mini-implants highlighted the different methods of force application using hooks with different heights^22^.

#### Force systems for simultaneous distalization and intrusion

During distalization of the entire maxillary dentition for Class II correction, using mechanics with mini-implants in the IZC, due to the force passing bellow the Cr, anterior teeth tend to rotate clockwise, loose torque and extrude. Such extrusion intensifies overbite, a welcome result should the patient have anterior open bite. On the other hand, in case there already is overbite, especially with gingival smile, anterior intrusion with interradicular mini-implants and mini-implants in IZC would be an option. In order to balance the clockwise rotation effect of the maxillary occlusal plane and favor gingival smile correction, while promoting anchorage to anterior retraction, it was suggested that aside from two IZC mini-implants another two were to be installed between central and lateral incisors.^23^ They would countereffect the anterior extrusion, resulting in intrusion of the entire maxillary dentition and favoring gingival smile correction. Furthermore, another suggestion would be gingivectomy in the anterior area (crown lengthening) in order to improve gingival smile.^23^

**Figure 30:**
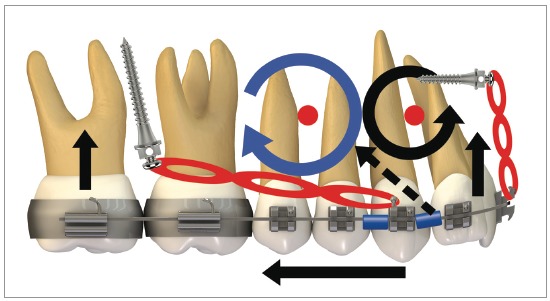
Sophisticated force system stemming from two mini-implants in the IZC and another two in the anterior area of maxilla: a dentoalveolar distalizing force of the entire maxillary dentition can be observed, due to the anchorage in the IZC. A clockwise rotation (moment) of the entire arch occurs around the maxilla's Cr. Intrusive vertical force occurs in the posterior area. A intrusive vertical force can be seen upon the incisors as well as a counterclockwise moment around the anterior teeth's Cr.

The diagram in Figure 30 shows the complex force system created by four mini-implants on the maxilla. A dentoalveolar distalization force of the entire maxillary arch can be observed, resulting from anchorage in the IZC. A clockwise rotation (moment) of the entire arch occurs around the maxilla's Cr. The vertical arrow in Figure 30 indicates an intrusive vertical force in the maxillary posterior area. An intrusive vertical force over the incisors and a counterclockwise moment around the Cr of maxillary anterior teeth can also be observed.

**Figure 31:**
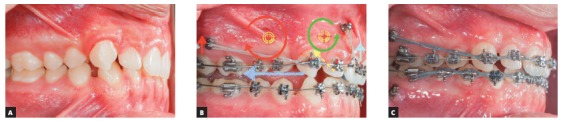
A) Young patient with Class II, division 2, malocclusion, overbite and gingival smile. B) Mechanics for distalizing the entire maxillary dentition with two mini-implants in the IZC, located between the first and second maxillary molars, and associated anterior intrusion, with two anterior mini-implants. C) Mechanics lasted for 12 months, after which an improvement in the sagittal arches relationship (Class I molars) could be observed.

In Figure 31, since the patient had Class II, division 2 malocclusion, with overbite and gingival smile, the treatment consisted of incisors intrusion along with distalization of the entire maxillary arch. The intrusion mechanics system was created for simultaneous intrusion and buccal movement of the incisors, in order to improve their axial inclinations. A 40g/side force for anterior intrusion was applied by means of chain elastics. Anterior mini-implants were positioned in front of the anterior teeth's center of resistance, generating a counterclockwise moment. To distalize the entire maxillary dentition, two IZC mini-implants were adapted between the first and second maxillary molars. Two stops, mesial and distal to the canines, locked the entire arch and chain elastics (e-chain, TP Orthodontics) were used for arch retraction, generating 300g/side force. The oblique component of force resultant caused maxillary intrusion. The anterior intrusion mechanics lasted for 12 months, after which an improvement in the sagittal arch relation (Class I molars) was observed.

## CONCLUSION

It is undeniable that extra-alveolar mini-implants anchorage has revolutionized Orthodontics. In the same way, the correct understanding of mini-implants Biomechanics allowed a wider range of dental movements, as never seen before in clinical practice. Thus, it is important to consider the many possibilities in force application systems stemming from skeletal anchorage use, in order to producing increasingly efficient treatments.
